# Cumulative Soil Metal Contamination Reshapes Oxidative and Neuroenzymatic Stress Responses in Ants Across an Industrial Pollution Gradient

**DOI:** 10.3390/life16050743

**Published:** 2026-04-29

**Authors:** Lucia-Florina Popovici, Silviu Giorgian Țicu, Ionela Ramona Zgavarogea, Lucian Hrițcu, Lăcrămioara Oprică, Ion Brînza, Ioan Tăușan

**Affiliations:** 1 Department of Agricultural Sciences and Food Engineering, “Lucian Blaga” University of Sibiu, 7–9 Ion Ratiu Street, 550024 Sibiu, Romania; luciaflorina.popovici@ulbsibiu.ro; 2 Romanian Academy of Scientists (AOSR), 030167 Bucharest, Romania; silviu-giorgian.ticu@s.unibuc.ro (S.G.Ț.); ioan.tausan@ulbsibiu.ro (I.T.); 3 Doctoral School of Biology, Faculty of Biology, University of Bucharest, Splaiul Independenței 91–95, 050095 Bucharest, Romania; 4 Natural History Museum, Brukenthal National Museum, Cetății 1, 550163 Sibiu, Romania; 5 National Institute for Research and Development for Cryogenic and Isotopic Technologies—ICSI, 240050 Râmnicu Vâlcea, Romania; ramona.zgavarogea@icsi.ro; 6 Faculty of Biology, Alexandru Ioan Cuza University, 20A Carol I Boulevard, 700506 Iasi, Romania; hritcu@uaic.ro (L.H.); lacramioara.oprica@uaic.ro (L.O.); 7 Department of Environmental Sciences, Biology and Ecology Research Center, Faculty of Sciences, “Lucian Blaga” University of Sibiu, Doctor. Ion Rațiu Street No. 5–7, 550012 Sibiu, Romania

**Keywords:** ants, soil metal contamination, oxidative stress, acetylcholinesterase, biomarkers, biomonitoring, pollution gradient

## Abstract

Metal(loid) contamination is a persistent environmental stressor in terrestrial ecosystems, yet field-based evidence linking cumulative soil contamination to physiological responses in social insects remains limited. In this study, we investigated an industrial pollution gradient by measuring soil concentrations of potentially toxic elements across multiple sites and integrating multi-element exposure into a cumulative pollution index. Two ant taxa, *Lasius niger* (Linnaeus, 1758) and *Tetramorium* cf. *caespitum* (Linnaeus, 1758), were sampled using a standardized field design, and biochemical endpoints were assessed to characterize antioxidant defense, thiol-based redox status, oxidative damage, and neuroenzymatic responses. Ant homogenates were analyzed spectrophotometrically for antioxidant enzymes, reduced glutathione, lipid peroxidation, protein oxidation, and acetylcholinesterase activity compared with the local low-contamination reference site. In addition, *PLI* showed positive site-level associations with multiple biomarkers, suggesting coordinated covariation between cumulative soil contamination and biochemical responses. Because these analyses were based on site-level mean values and direct tissue metal burdens were not measured, the findings should be interpreted as field-based associations rather than evidence of direct internal dose–response or metal-specific causality. These findings suggest that cumulative soil metal(loid) contamination is linked to integrated oxidative and neuroenzymatic stress responses in ants and support the use of ant-based biomarkers as informative tools for ecological biomonitoring under field conditions.

## 1. Introduction

Metal(loid) contamination represents one of the most persistent anthropogenic pressures on terrestrial ecosystems, as, as potentially toxic elements are non-biodegradable, accumulate in soil, and may remain bioavailable over long time scales, thereby influencing the structure and functioning of edaphic biocenoses [1,2,3]. At the European level, harmonized assessments of topsoil indicate that metalloids such as arsenic (As), together with heavy metals such as cadmium (Cd), copper (Cu), mercury (Hg), lead (Pb), zinc (Zn), nickel (Ni), and cobalt (Co) can reach ecotoxicologically relevant concentrations in certain regions, with direct implications for ecosystem health and trophic transfer risks [3]. Consequently, the integrated monitoring of metal burden and associated biological effects has become a priority for environmental management and for elucidating sublethal mechanisms that precede population decline [4].

Among terrestrial invertebrates, ants (Hymenoptera: Formicidae) are regarded as useful sentinels of environmental disturbance due to their abundance, ecological importance, broad distribution, and intimate contact with the soil environment through nesting, excavation, foraging, and trophic interactions [5,6]. Their colonial organization and relatively stable occurrence across habitats further support their use in biomonitoring studies, where they can integrate local exposure over ecologically relevant spatial scales [5,6,7]. In addition to community-based metrics, biomarker approaches can provide complementary insight by detecting sublethal physiological stress before more evident ecological effects emerge [5]. In the present study, we focused on *Lasius niger* (Linnaeus, 1758) and *Tetramorium* cf. *caespitum* (Linnaeus, 1758), hereafter referred to as *L. niger* and *T.* cf. *caespitum*, two widespread and ecologically relevant ant taxa commonly found in anthropogenic habitats. Although both are predominantly epigeic and interact closely with the soil surface, they differ in functional and ecological traits such as foraging strategy, microhabitat use, and tolerance to environmental heterogeneity, which makes them suitable for evaluating biomarker responses under shared but not necessarily identical exposure contexts [7,8,9,10].

A central mechanism through which continuous metal exposure exerts sublethal toxicity is the disruption of redox homeostasis. Metals may catalyze pro-oxidant reactions, interact with thiol groups, impair mitochondrial function, and affect antioxidant enzymes, thereby promoting the accumulation of reactive oxygen species (ROS) and the onset of oxidative stress [11,12]. From this perspective, antioxidant biomarkers such as superoxide dismutase (SOD), catalase (CAT), and glutathione peroxidase (GPx) are interpreted either as compensatory responses limiting oxidative damage or as a “signal” of chronic stress when their activation persists concomitantly with increased markers of injury (e.g., lipid peroxidation and protein oxidation) [13]. However, interpretation of these responses in field conditions remains challenging. Increased antioxidant activity may reflect successful compensation, but it may also indicate sustained physiological cost when accompanied by elevated damage markers. Moreover, in natural systems, redox-related biomarkers are influenced not only by contaminants but also by multiple co-occurring abiotic and biotic factors, including microclimatic variation, trophic resource availability, colony demography and social organization, seasonal context, and biological stressors such as pathogens, parasites, or other microorganism-mediated interactions [14,15,16]. Previous studies suggest that metal(loid) exposure in ants may affect not only bioaccumulation patterns and colony performance, but also components of innate immunity. In a Finnish population of the red wood ant *Formica aquilonia*, Sorvari et al. [17] showed that industrial metal pollution disturbed the encapsulation response, supporting the view that chronic contamination may impair immune defense in free-living ants. Other studies from Finland further reported elevated body metal burdens together with reduced nest size or altered colony-level traits in polluted environments [15,18], while work from Italy [19,20] and Poland [21] expanded this perspective by showing metal accumulation in ant tissues, altered metal tolerance, and interactions between ant nests and microbial communities in contaminated soils. Together, these studies indicate that the biological effects of metal pollution in ants extend beyond simple accumulation and may involve immune, physiological, and microbe-associated pathways that should be considered when interpreting biomarker responses under field conditions.

Beyond oxidative stress, contaminants may also affect neurophysiological function. Acetylcholinesterase (AChE), a key enzyme involved in cholinergic neurotransmission, is widely used in ecotoxicology as a biomarker of sublethal neurotoxicity [22]. Under environmentally realistic exposure scenarios, AChE activity may be modulated by direct contaminant effects, indirect redox-related protein alterations, or broader physiological dysregulation associated with chronic stress [11,12,22]. For this reason, combining oxidative stress markers with neuroenzymatic endpoints may offer a more integrated view of organismal response than any single biomarker alone. Nevertheless, despite growing interest in ant-based biomonitoring, evidence linking cumulative soil metal pressure to coordinated multimarker responses in ants remains limited, especially under field conditions characterized by long-term industrial contamination and mixed exposure profiles.

Because free-living organisms are typically exposed to contaminant mixtures rather than isolated metals, cumulative indices may provide a more ecologically realistic representation of environmental pressure than single-element assessments alone. The Pollution Load Index (*PLI*), originally proposed as an integrative measure of multi-element contamination, is particularly useful in this regard because it summarizes contamination factors across metals and allows direct comparison among sites along a gradient [4]. This type of approach is especially relevant in historically industrialized areas, where contamination is spatially heterogeneous and often reflects the combined legacy of long-term emissions rather than a single source or a single pollutant. The Copșa Mică region in Romania is one such area, having a documented history of industrial metal pollution and persistent concern regarding soil contamination [23]. Importantly, within such a landscape, local reference sites should be interpreted cautiously. Rather than representing pristine background conditions, they may more appropriately function as comparatively lower-contamination sites within a broader region with historical environmental pressure.

In this context, the present study examined whether cumulative soil metal(loid) contamination is associated with integrated oxidative and neuroenzymatic responses in ants distributed along an industrial pollution gradient. We analyzed two ant taxa, *L. niger* and *T.* cf. *caespitum*, across sites differing in multi-element soil contamination and related a cumulative pollution index to biomarkers of antioxidant defense, thiol-based redox balance, oxidative damage, and AChE activity. We hypothesized that increasing cumulative soil contamination would be associated with coordinated biomarker shifts, consistent with field-based physiological stress responses. By focusing on field-based pressure–response associations rather than on a simple polluted-versus-pristine contrast, this study aims to support the potential use of ant biochemical markers as ecologically informative tools for biomonitoring in metal(loid)-impacted terrestrial environments, while acknowledging the inherent complexity and potential confounding influences of natural field systems.

## 2. Materials and Methods

### 2.1. Design and Research Area

This study was designed as a field-based comparative investigation conducted along a spatial gradient of soil metal(oid) contamination in Sibiu County, Romania. The main objective was to examine whether cumulative soil metal contamination was associated with variation in oxidative and neuroenzymatic biomarkers in ants. To ensure comparability across locations and between taxa, the sampling design and analytical workflow were standardized for both investigated ant taxa, *L. niger* and *T.* cf. *caespitum.*

**Figure 1:**
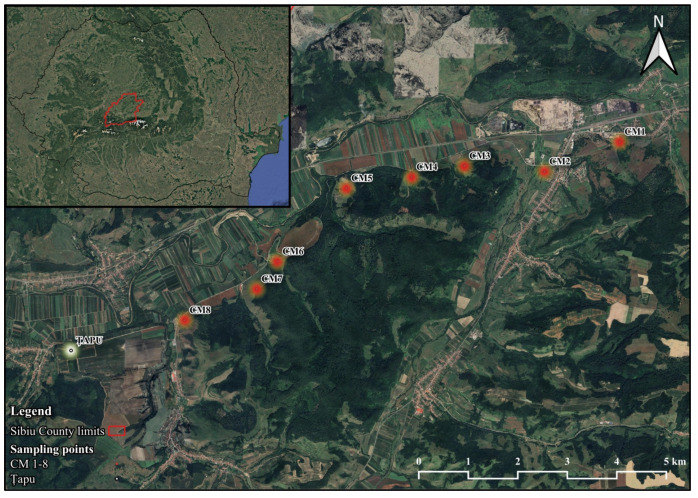
Geographic location of the sampling sites in the Copșa Mică–Țapu area (Sibiu County, Romania). The satellite map illustrates the spatial distribution of the study sites along a soil metal(oid) contamination gradient. Sites located in the Copșa Mică industrial area are marked as C1–C8 and correspond to sampling points CM1–CM8 positioned in proximity to the historical industrial emission source. The local low-contamination reference site, Țapu (TAPU) (labeled C9 on the map) and was used as the background site for the calculation of the Pollution Load Index (*PLI*). The exact geographic coordinates (latitude and longitude). Basemap source: Google. (2025). [Map of Sibiu County l]. Retrieved 28 April 2025, from google.

Field sampling was performed in the Copșa Mică–Micăsasa–Țapu area, a region historically affected by industrial emissions associated with metallurgical activity. Eight sampling sites were selected in the Copșa Mică area (CM1–CM8), representing the contamination gradient [24], and one additional site in Țapu (TAPU) was included as a local low-contamination reference site within the study landscape. The approximate distance between Copșa Mică and TAPU was 11.8 km, as estimated using Google Maps Google. (2025). [Map of Sibiu County l]. Retrieved 28 April 2025, from google. The spatial distribution of sampling sites is shown in Figure 1.

The geographic coordinates of the sampling sites were as follows: CM1: 46.1132° N, 24.2423° E; CM2: 46.1077° N, 24.2228° E; CM3: 46.1085° N, 24.2018° E; CM4: 46.1064° N, 24.1881° E; CM5: 46.1043° N, 24.1711° E; CM6: 46.0908° N, 24.1533° E; CM7: 46.0858° N, 24.1479° E; CM8: 46.0799° N, 24.1292° E; TAPU: 46.0742° N, 24.0996° E).

For each species, three distinct colonies were sampled at each site and considered independent biological replicates. From each colony, three ant subsamples were collected, yielding a total of nine ant samples per site for each species. For statistical analysis, the subsamples obtained from the same colony were treated as colony-level subsamples. Colonies were selected from distinct nest entrances or microhabitats within the same site in order to capture within-site variability and avoid repeated sampling of the same nest structure. The same sampling strategy was applied at the TAPU site. The geographic positions of each colony were recorded using a portable GPS device (GPSMAP 64s, Garmin International, Inc., Olathe, KS, USA) to ensure sampling traceability and facilitate future replication.

### 2.2. Soil Sampling and Metal(oid) Analysis

#### 2.2.1. Soil Sampling

At each sampling site, three soil cores were collected as site-level replicates from the immediate vicinity of the sampled ant colonies. Soil was sampled to a depth of 15 cm using a corer with a diameter of 10 cm. Sample collection, packaging, transport, storage, and preservation were performed in accordance with internationally harmonized soil-sampling guidance and the corresponding ISO standards (ISO 18400-105:2017; ISO 18400-104:2018) [24,25].

The concentrations of selected potentially toxic elements in soil were determined for cadmium (Cd), chromium (Cr), cobalt (Co), copper (Cu), lead (Pb), manganese (Mn), nickel (Ni), zinc (Zn), arsenic (As), and mercury (Hg), together with the additional elements included in the analytical panel where applicable. Measured soil metal(oid) concentrations were interpreted according to the Romanian Regulation on Environmental Pollution Assessment, approved by Order No. 756/1997, using the alert and intervention thresholds specified in the annex for sensitive and less sensitive land-use categories [26].

#### 2.2.2. Apparatus and Reagents

Elemental analyses were performed using an inductively coupled plasma mass spectrometer (ICP-MS 820-MS, Varian, Australia Pty Ltd., Mulgrave, VIC, Australia) equipped with an SPS-3 autosampler, a micro-concentric nebulizer, nickel cones, and a peristaltic pump for sample introduction, all supplied as components of the ICP-MS system. Argon gas of 6.0 purity (Messer, Austria) was used for plasma generation.

Microwave digestion was carried out using a Mars 5 microwave digestion system (CEM Microwave Technology Ltd., Buckingham, UK) equipped with 100 mL Teflon digestion vessels. Calibration standards were prepared from ICP Multi Element Standard Solution XXI CertiPUR (Merck, Darmstadt, Germany). Reagents used for sample preparation included nitric acid (HNO3, 69%, *w*/*v*), hydrofluoric acid (HF), hydrochloric acid (HCl), and ultrapure water (18.2 MΩ·cm) obtained from a Milli-Q Millipore system (Bedford, MA, USA). The instrumental and data acquisition parameters used for ICP-MS measurements are presented in Table 1. Analytical quality control included calibration with multi-element standard solutions, procedural blanks, duplicate measurements, and verification of analytical accuracy using certified reference material, where available. Recovery values within 80–110% were considered acceptable for the investigated elements. These quality-control steps were used to monitor contamination during sample preparation, instrumental stability, and the reliability of the measured elemental concentrations.

Before analysis, all samples were carefully prepared using standardized handling procedures to minimize contamination, matrix effects, and sample loss.

#### 2.2.3. Soil Sample Preparation (Microwave Digestion)

Soil samples were air-dried, homogenized, and passed through a 20-mesh sieve prior to digestion. Microwave-assisted acid digestion was performed using a previously optimized protocol. Briefly, 0.25 g of soil was placed in a Teflon digestion vessel together with 9 mL of 65% HNO_3_, 3 mL of concentrated HF, and 2 mL of concentrated HCl. The closed vessels were digested in the microwave system by increasing the temperature to 200 °C over 6 min and maintaining this temperature for 20 min. The digestion program is presented in Table 2.

After cooling, the digests were transferred quantitatively into 100 mL volumetric flasks and brought to volume with ultrapure water. The resulting solutions were analyzed by ICP-MS after appropriate dilution.

#### 2.2.4. ICP-MS Analysis

Elemental concentrations were determined by multi-element ICP-MS analysis using external standard calibration. Calibration standards were prepared at five concentration levels (2.5, 5, 10, 25, and 50 mg/L) from the certified multi-element stock solution. Each sample was analyzed in duplicate, and each analytical run included five replicate readings. Calibration standards and duplicate measurements were used to monitor analytical precision and instrumental stability during the analytical sequence.

#### 2.2.5. Method Validation and Quality Control

Analytical accuracy was evaluated using certified reference material (SRM NCS ZC 73006; soil composition including trace elements), which was processed under the same digestion and analytical conditions as the soil samples, in accordance with ICP-MS method-validation procedures previously described for metal determination in soils [27]. Recovery values ranged between 80% and 110% were considered acceptable for the investigated elements, indicating satisfactory agreement with the certified reference values. Duplicate analyses were used to assess analytical repeatability, while calibration standards were used to verify instrument response during quantification. These quality-control procedures were applied to improve confidence in the measured elemental concentrations.

### 2.3. Pollution Load Index (PLI) and Soil Contamination Assessment

To integrate the multi-element contamination data into a single comparative metric, a Pollution Load Index (*PLI*) was calculated for each sampling site. The *PLI* approach allows the assessment of cumulative metal(oid) contamination relative to the local low-contamination reference site and reduces the need to interpret multiple individual element comparisons separately.

First, the Contamination Factor (*CF_i_*) for each metal was calculated according to:

$$CF_{i}=\frac{C_{i}}{C_{ref}}$$

where $C_{i}$ represents the measured concentration (mg/kg dry weight) of metal *i* at a given site, and $C_{ref}$ corresponds to the concentration of the same element measured at TAPU, the local low-contamination reference site used as the baseline for this study.

To improve biological relevance, the primary *PLI* used for biomarker correlations was calculated for elements with documented toxicological significance and oxidative or neurotoxic potential in invertebrates, namely: Pb, Cd, Zn, Cu, Ni, As and Hg. The site-specific *PLI* was then computed as the geometric mean of the selected contamination factors:

$$PLI=(CF_{Pb}\times CF_{Cd}\times CF_{Zn}\times CF_{Cu}\times CF_{Ni}\times CF_{As}\times CF_{Hg}{)}^{1/7}$$

A *PLI* value > 1 indicates higher cumulative relative to the local reference baseline, whereas *PLI* < 1 suggests lower contamination relative to that baseline. This integrated soil contamination index was subsequently used in site-level correlation analyses with biochemical biomarkers, including SOD, CAT, GPx, GSH, MDA, PC, and AChE, to evaluate associations between cumulative soil metal(loid) contamination and oxidative or neuroenzymatic biomarker variation.

### 2.4. Determination of Biochemical Parameters

After collection, worker ants were transported to the laboratory, weighed (~3 g per batch), aliquoted into 2 mL microtubes, and stored at −80 °C until analysis. Samples were processed on ice. Ants were homogenized in 0.1 M potassium phosphate buffer (pH 7.4) containing 1.15% KCl and centrifuged at 14,000 rpm for 10 min at 4 °C. The supernatant was used for biochemical assays. Spectrophotometric readings were performed using a UV–Vis spectrophotometer (DU 730, Beckman Coulter, Inc., Brea, CA, USA) as previously described [28,29]. Protein concentration was determined using the Bradford method [30], with bovine serum albumin (BSA) as standard. Enzymatic activities were normalized to total protein concentration and expressed as specific activity (U/mg protein). All determinations were performed in technical triplicate.

#### 2.4.1. Superoxide Dismutase (SOD) Activity Assay

SOD activity was determined using the nitroblue tetrazolium (NBT) photoreduction method, which is based on the inhibition of NBT reduction in the presence of superoxide radicals generated by the riboflavin–light system [31]. The reaction mixture contained 0.067 M phosphate buffer (pH 7.8), 0.1 M EDTA, 1.5 mM NBT, 0.12 mM riboflavin, and a defined volume of enzymatic extract. Samples were exposed to fluorescent light for a standardized interval, and absorbance was measured at 560 nm against a blank. SOD activity was expressed as enzymatic units and normalized to protein concentration (U/mg protein).

#### 2.4.2. Catalase (CAT) Activity Assay

CAT activity was assessed by quantifying hydrogen peroxide (H_2_O_2_) decomposition using the dichromate–acetic acid method [32]. The enzymatic extract was incubated with an H_2_O_2_ substrate solution for a fixed time, and the reaction was stopped by adding the dichromate–acetic acid reagent. The mixture was heated at 95 °C, centrifuged, and the absorbance of the supernatant was read at 570 nm. CAT activity was calculated from the difference between the initial H_2_O_2_ amount (blank) and the residual H_2_O_2_ after reaction and expressed as specific activity (U/mg protein).

#### 2.4.3. Glutathione Peroxidase (GPx) Activity Assay

GPx activity was determined by measuring the consumption of reduced glutathione (GSH) in the presence of H_2_O_2_ [33]. Extracts were incubated in phosphate buffer in the presence of EDTA and sodium azide; the reaction was initiated by adding GSH and H_2_O_2_ and stopped after a standardized interval by adding 7% metaphosphoric acid. After centrifugation, the supernatant was reacted with DTNB, and absorbance was measured at 412 nm. GPx activity was expressed as µmol GSH oxidized/min and normalized to protein concentration (U/mg protein).

#### 2.4.4. Reduced Glutathione (GSH) Level

GSH levels were quantified based on the reaction of thiol groups with 5,5′-dithiobis(2-nitrobenzoic acid) (DTNB), yielding the chromophore 5-thio-2-nitrobenzoate (TNB), measured spectrophotometrically [34]. Reactions were performed in 1.5 mL microtubes using 0.3 M Na_2_HPO_4_ and 0.04% DTNB, under three conditions: sample (homogenate + DTNB), control (without homogenate), and sample control (homogenate without DTNB). Absorbance was read at 412 nm at 2 min after reaction initiation using a UV–Vis spectrophotometer (Beckman Coulter DU 730) in plastic cuvettes. Values were blank-corrected and normalized to protein content (mg protein).

#### 2.4.5. Lipid Peroxidation (MDA)

MDA, as an indicator of lipid peroxidation, was determined using the TBARS (thiobarbituric acid reactive substances) assay [35]. Extracts were incubated with TBA reagent at 95 °C for a standardized duration, cooled, and centrifuged. Absorbance was measured at 532 nm. MDA concentration was expressed as MDA equivalents and normalized to protein content (e.g., nmol MDA/mg protein).

#### 2.4.6. Protein Carbonyl Content (PC)

PC was used as a marker of protein oxidation and was quantified via reaction of carbonyl groups with 2,4-dinitrophenylhydrazine (DNPH) [36]. After hydrazone derivative formation, absorbance was measured at 370 nm. Protein carbonyl content was expressed as nmol carbonyl/mg protein.

#### 2.4.7. Acetylcholinesterase (AChE) Activity Assay

AChE activity was determined using the Ellman method [37], with AChE iodide as substrate and DTNB as the chromogenic reagent for detection of the produced thiocholine. The reaction was incubated at 37 °C for a standardized interval, and absorbance was read at 412 nm. AChE activity was expressed as nmol substrate hydrolyzed/min and normalized to protein concentration (U/mg protein).

### 2.5. Statistical Analysis

Statistical analyses were performed using GraphPad Prism (v9.0). Data are presented as mean ± SD, and statistical significance was set at *p* < 0.05. The two ant taxa, *L. niger* and *T.* cf. *caespitum*, were analyzed separately. Colonies were treated as a unit of biological replication. For each site and species, three colonies were sampled and analyzed as independent biological replicates. From each colony, three subsamples were processed independently, and for each subsample, three technical determinations were performed. The mean of the technical replicates was first calculated for each subsample, and the mean of the three subsamples was then used as the single colony-level value for statistical analysis.

Data normality was assessed using the Shapiro–Wilk test. Site-wise differences in biomarker levels were analyzed using one-way ANOVA followed by Tukey’s multiple-comparisons test. These analyses were used to describe spatial variation in biochemical profiles among sampling sites. Because the primary objective of the study was to evaluate field-based pressure–response associations along a continuous contamination gradient, the main inferential analyses were based on site-level associations between the Pollution Load Index (*PLI*) and biochemical biomarkers. For this purpose, colony-level values were averaged within each site to obtain one site-level mean per biomarker and species, and correlations between *PLI* and biomarker values were assessed using Spearman’s rank correlation test (two-tailed, exact *p* values).

To further explore the internal coherence of the oxidative stress response, additional Spearman correlations were calculated between malondialdehyde (MDA) and the other biochemical parameters at the site level. Because these correlations were calculated using site-level mean values, the number of independent observations was limited. Therefore, very strong correlations were interpreted as evidence of coordinated covariation rather than as definitive estimates of effect magnitude.

Because internal metal burdens were not measured directly in ant tissues, the statistical associations between soil *PLI* and biomarker responses were not interpreted as direct internal dose–response relationships, but rather as field-based associations between cumulative soil metal(loid) contamination and oxidative or neuroenzymatic biomarker variation.

### 2.6. Ethical Considerations

The study involved invertebrate species (*L. niger* and *T.* cf. *caespitum*). According to national legislation, no specific ethical approval was required for research on invertebrates. Sample collection was conducted in compliance with local environmental protection regulations.

## 3. Results

### 3.1. Soil Metal(loid) Profile and Integrated Pollution Load

**Figure 2:**
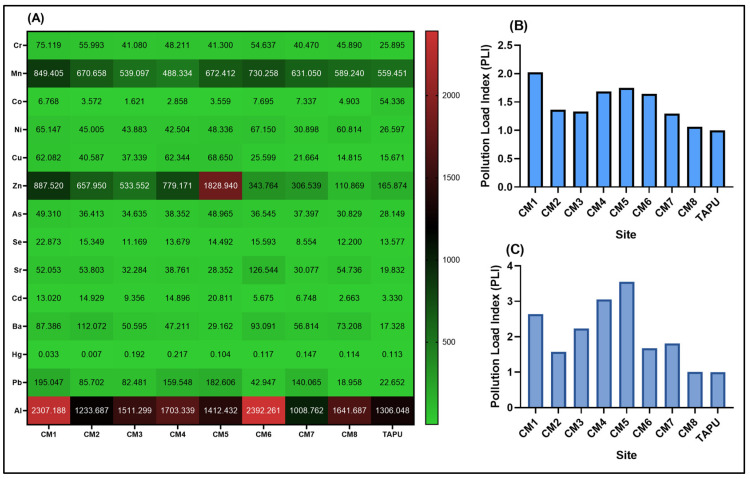
(**A**) Heatmap showing site-level concentrations of arsenic (As), cadmium (Cd), cobalt (Co), chromium (Cr), copper (Cu), iron (Fe), mercury (Hg), manganese (Mn), nickel (Ni), lead (Pb), selenium (Se), strontium (Sr), vanadium (V), and zinc (Zn), expressed as mg/kg dry weight, at sites CM1–CM8 and at the local reference site, Țapu (TAPU). (**B**) Pollution Load Index (*PLI*) calculated at the site level using TAPU as the local low-contamination reference and including all 14 quantified elements. (**C**) *PLI* calculated at the site level using TAPU site, based on a toxicologically prioritized subset of seven elements, Pb, Cd, Zn, Cu, Ni, As, and Hg.

The analysis of soil elemental composition showed a clear spatial variation across the across the Copșa Mică sites (CM1–CM8), with higher multi-element burdens than at the local low-contamination reference site, TAPU (Figure 2A). These data defined a relative soil contamination gradient within a historically impacted landscape.

To contextualize these findings within the Romanian regulatory framework, measured concentrations were interpreted according to Order No. 756/1997 [26] on environmental pollution assessment, which defines normal values together with alert and intervention thresholds for sensitive and less sensitive land uses (Table 3). The threshold values relevant to the present study are summarized below. In cases of uncertainty regarding land-use classification, the regulation indicates that the thresholds for sensitive land use should be considered.

Classification of site concentrations according to these thresholds showed substantial exceedances for several elements (Table 4). Arsenic exceeded the intervention threshold at all sites, including TAPU, indicating a widespread regional burden. Cadmium exceeded the intervention threshold at most Copșa Mică sites, while zinc reached intervention levels at CM1, CM4, and CM5. Lead frequently exceeded the alert threshold at Copșa Mică sites, consistent with a substantial contribution from historical industrial emissions. These threshold-based classifications provide an environmental context for the gradient identified by the *PLI* calculations.

Although TAPU was used as the local low-contamination reference site for relative comparisons, its profile was not fully free of exceedances, as arsenic and selenium also reached elevated categories at this site. This indicates that the reference condition should be interpreted cautiously, likely reflecting either regional atmospheric deposition or local geochemical background.

Overall, the soil data describe a cumulative metal(loid) contamination gradient across the study area and provide the geochemical basis for subsequent site-level comparisons with ant biochemical biomarkers.

### 3.2. Antioxidant Enzymatic Response

**Figure 3:**
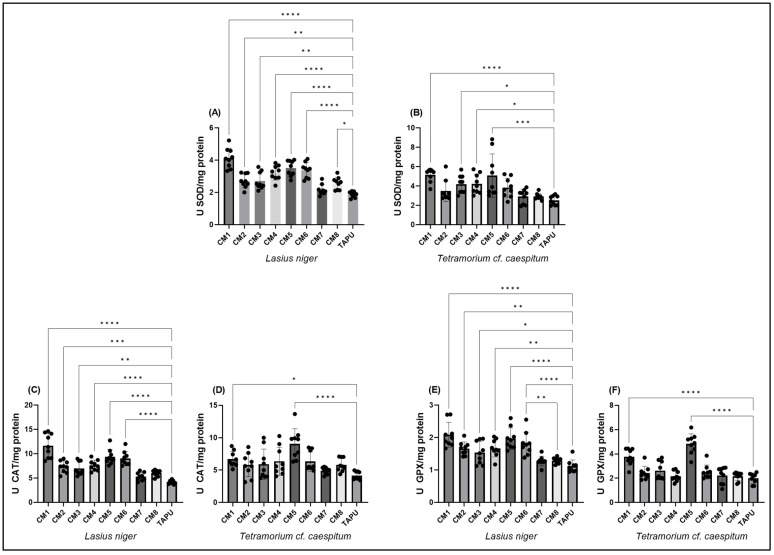
Antioxidant enzymatic response in *L. niger* and *T.* cf. *caespitum* across sampling sites. (**A**,**B**) Superoxide dismutase (SOD) activity; (**C**,**D**) catalase (CAT) activity; (**E**,**F**) glutathione peroxidase (GPx) activity measured in *L. niger* (**A**,**C**,**E**) and *T.* cf. *caespitum* (**B**,**D**,**F**). Data are presented as mean ± SD based on three colonies per site and species, with three subsamples analyzed per colony. CM1–CM8 represent sampling sites from the Copșa Mică industrial area, and TAPU represents the local low-contamination reference site within the study landscape. Statistical differences among sites were assessed using one-way ANOVA followed by Tukey’s multiple comparisons test. Significance coding: * *p* < 0.05; ** *p* < 0.01; *** *p* < 0.001; **** *p* < 0.0001. Complete Tukey-adjusted post hoc comparison results are reported in Supplementary Table S1.

To assess the enzymatic antioxidant profiles across the study sites, SOD, CAT, and GPx activities were measured in both species. As illustrated in Figure 3A–F, several Copșa Mică sites showed higher antioxidant enzyme activities compared with TAPU, with different spatial patterns between *L. niger* and *T.* cf. *caespitum*.

SOD activity differed significantly among sites in both species. In *L. niger*, SOD activity showed a strong site effect (one-way ANOVA: F(8,72) = 22.72, *p* < 0.0001; R^2^ = 0.716), with significantly higher values at most Copșa Mică sites compared with TAPU, except CM7. The highest SOD activity was observed at CM1 (Figure 3A; Supplementary Table S1). In *T.* cf. *caespitum*, SOD activity also varied significantly among sites (F(8,72) = 6.878, *p* < 0.0001; R^2^ = 0.433), but the response was more restricted, with significant increases mainly at CM1, CM3, CM4, and CM5 relative to TAPU (Figure 3B; Supplementary Table S1). Variance heterogeneity was detected for *T.* cf. *caespitum*, SOD data, and finer site-level contrasts should therefore be interpreted with caution.

CAT activity also displayed significant site-dependent variation in both taxa. In *L. niger*, CAT activity differed strongly among sites (F(8,72) = 25.58, *p* < 0.0001; R^2^ = 0.740), with increased activity at CM1–CM6 relative to TAPU, whereas CM7 and CM8 were closer to reference levels (Figure 3C; Supplementary Table S1). In *T.* cf. *caespitum*, CAT activity varied significantly among sites as well (F(8,72) = 6.042, *p* < 0.0001; R^2^ = 0.402), but the increase was more localized, being significant mainly at CM1 and especially CM5 compared with TAPU (Figure 3D; Supplementary Table S1).

GPx activity followed a similar pattern of site-dependent modulation. In *L. niger*, GPx differed significantly among sites (F(8,72) = 12.77, *p* < 0.0001; R^2^ = 0.587), with significantly higher activity at CM1–CM6 relative to TAPU, while CM7 and CM8 did not differ from the reference site (Figure 3E; Supplementary Table S1). In *T.* cf. *caespitum*, GPx also showed a strong site effect (F(8,72) = 22.07, *p* < 0.0001; R^2^ = 0.710), characterized by marked increases at CM1 and CM5, with CM5 representing the most pronounced response (Figure 3F; Supplementary Table S1).

Overall, SOD, CAT, and GPx activities showed significant site-dependent variation in both taxa. *L. niger* displayed higher antioxidant enzyme activities across a broader set of Copșa Mică sites, whereas *T.* cf. *caespitum* showed a more localized pattern, mainly involving CM1 and/or CM5.

### 3.3. The Glutathione System

**Figure 4:**
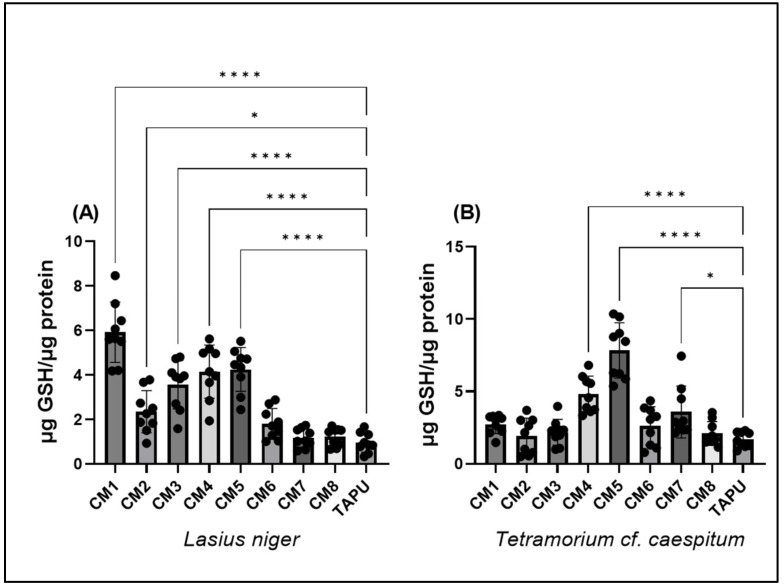
Reduced glutathione (GSH) levels in *L. niger* and *T.* cf. *caespitum* across sampling sites. (**A**) GSH in *L. niger*; (**B**) GSH in *T.* cf. *caespitum.* Data are presented as mean ± SD based on three colonies per site and species, with three subsamples analyzed per colony. CM1–CM8 represent sampling sites from the Copșa Mică industrial area, and TAPU represents the local low-contamination reference site within the study landscape. Statistical differences among sites were assessed using one-way ANOVA followed by Tukey’s multiple comparisons test. Significance coding: * *p* < 0.05; **** *p* < 0.0001. Complete Tukey-adjusted post hoc comparison results are reported in Supplementary Table S2.

GSH levels were measured to evaluate site-dependent variation in thiol-related antioxidant status. In both *L. niger* and *T.* cf. *caespitum*, GSH levels showed significant spatial variation among sampling sites (Figure 4).

In *L. niger*, GSH levels differed significantly among sites (one-way ANOVA: F(8,72) = 33.13, *p* < 0.0001; R^2^ = 0.786; Figure 4A). Compared with TAPU, GSH levels were significantly higher at CM1–CM5, whereas CM6–CM8 did not differ from the reference site. The highest values were observed at CM1 (Figure 4A; Supplementary Table S2).

In *T.* cf. *caespitum*, GSH levels also varied significantly across sites (F(8,72) = 22.57, *p* < 0.0001; R^2^ = 0.715), but the spatial pattern differed from that observed in *L. niger*. The response was dominated by pronounced increases at CM4 and especially CM5, while CM7 showed only a slight increase relative to TAPU. No significant differences from TAPU were observed at CM1–CM3, CM6, or CM8 (Figure 4B; Supplementary Table S2). Variance heterogeneity was detected for this dataset, and marginal site-level contrasts should therefore be interpreted cautiously.

Overall, GSH profiles differed between taxa: *L. niger* showed higher values across several Copșa Mică sites, whereas *T.* cf. *caespitum* displayed a more localized pattern, with a marked peak at CM5.

### 3.4. Oxidative Stress Biomarkers: Protein Oxidation and Lipid Peroxidation

**Figure 5:**
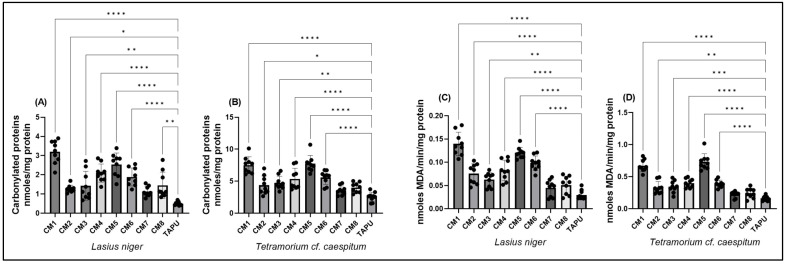
Oxidative damage biomarkers in *L. niger* and *T.* cf. *caespitum* across sampling sites. (**A**,**B**) Protein oxidation expressed as protein carbonyls (PC) in *L. niger* (**A**) and *T.* cf. *caespitum* (**B**). (**C**,**D**) Lipid peroxidation expressed as malondialdehyde (MDA) in *L. niger* (**C**) and *T.* cf. *caespitum* (**D**). Data are presented as mean ± SD based on three colonies per site and species, with three subsamples analyzed per colony. CM1–CM8 represent sampling sites from the Copșa Mică industrial area, and TAPU represents the local low-contamination reference site within the study landscape. Differences among sites were evaluated by one-way ANOVA followed by Tukey’s multiple comparisons test (adjusted *p*). Significance coding: * *p* < 0.05; ** *p* < 0.01; *** *p* < 0.001; **** *p* < 0.0001. Complete Tukey-adjusted post hoc comparison results are reported in Supplementary Table S3.

PC and MDA were measured to describe site-dependent variation in protein oxidation and lipid peroxidation, respectively. Both biomarkers showed spatial differences between Copșa Mică sites and TAPU in *L. niger* and *T.* cf. *caespitum* (Figure 5).

PC content differed significantly among sites in both species. In *L. niger*, PC showed a strong site effect (one-way ANOVA: F(8,72) = 22.93, *p* < 0.0001; R^2^ = 0.718), with significantly higher values at most Copșa Mică sites compared with TAPU. The response was particularly pronounced at CM1 and CM5, whereas CM7 did not differ from the reference site (Figure 5A; Supplementary Table S3). In *T.* cf. *caespitum*, PC also varied significantly among sites (F(8,72) = 19.86, *p* < 0.0001; R^2^ = 0.688), with maximal values at CM1 and CM5 and significant increases relative to TAPU at CM1–CM6. In contrast, CM7 and CM8 remained closer to reference levels (Figure 5B; Supplementary Table S3).

MDA levels showed a similar pattern of site-dependent oxidative damage. In *L. niger*, MDA differed markedly among sites (F(8,72) = 35.22, *p* < 0.0001; R^2^ = 0.796), with significant increases at CM1–CM6 compared with TAPU, while CM7 and CM8 were not significantly different from the reference site (Figure 5C; Supplementary Table S3). The highest values were observed at CM1 and CM5. In *T.* cf. *caespitum*, MDA displayed an even stronger site effect (F(8,72) = 51.52, *p* < 0.0001; R^2^ = 0.851), with the highest values again observed at CM1 and CM5. Significant increases relative to TAPU were also observed at CM2–CM6, whereas CM7 and CM8 did not differ from the reference site (Figure 5D; Supplementary Table S3).

Overall, PC and MDA profiles showed non-uniform spatial distributions, with the highest values generally observed at CM1 and CM5 and additional increases at several intermediate Copșa Mică sites.

### 3.5. Cholinergic Marker: Acetylcholinesterase (AChE) Activity

**Figure 6:**
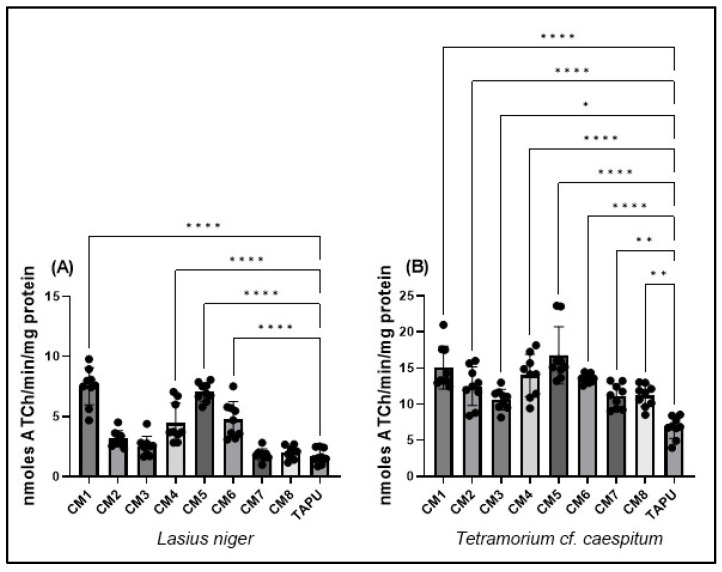
Acetylcholinesterase (AChE) activity in *L. niger* and *T.* cf. *caespitum* across sampling sites. (**A**) AChE activity in *L. niger*; (**B**) AChE activity in *T.* cf. *caespitum*. Data are presented as mean ± SD based on three colonies per site and species, with three subsamples analyzed per colony. CM1–CM8 represent sampling sites from the Copșa Mică industrial area, and TAPU represents the local low-contamination reference site within the study landscape. Statistical differences among sites were assessed using one-way ANOVA followed by Tukey’s multiple comparisons test. Significance coding: * *p* < 0.05; ** *p* < 0.01; **** *p* < 0.0001. Complete Tukey-adjusted post hoc comparison results are reported in Supplementary Table S4.

AChE activity was measured to describe site-dependent variation in the cholinergic biomarker profile. Measurements performed in *L. niger* and *T.* cf. *caespitum* revealed significant spatial differences among sites (Figure 6).

In *L. niger*, AChE activity differed significantly among sites (one-way ANOVA: F(8,72) = 39.50, *p* < 0.0001; R^2^ = 0.8144; Figure 6A). The highest activities were observed at CM1 and CM5, both markedly above TAPU, while CM4 and CM6 showed intermediate increases. In contrast, CM7 and CM8 were close to the reference site and did not differ significantly from TAPU (Figure 6A; Supplementary Table S4). Variance heterogeneity was detected for this dataset, and marginal site-level contrasts should therefore be interpreted cautiously.

In *T.* cf. *caespitum*, AChE activity also varied significantly among sites (one-way ANOVA: F(8,72) = 13.83, *p* < 0.0001; R^2^ = 0.6058; Figure 6B). AChE activity was significantly higher than TAPU at all Copșa Mică sites, with the strongest increases at CM5 and CM1 and similarly elevated values at CM4 and CM6 (Figure 6B; Supplementary Table S4). Although CM3, CM7, and CM8 showed lower values than the main high-response sites, they remained above the TAPU reference level.

Overall, AChE activity showed significant site-dependent variation in both taxa. *L. niger* displayed clear peaks at CM1 and CM5, whereas *T.* cf. *caespitum* showed higher AChE activity across all Copșa Mică sites compared with TAPU.

### 3.6. Correlation Analysis Between Pollution Load and Biochemical Biomarkers

**Figure 7:**
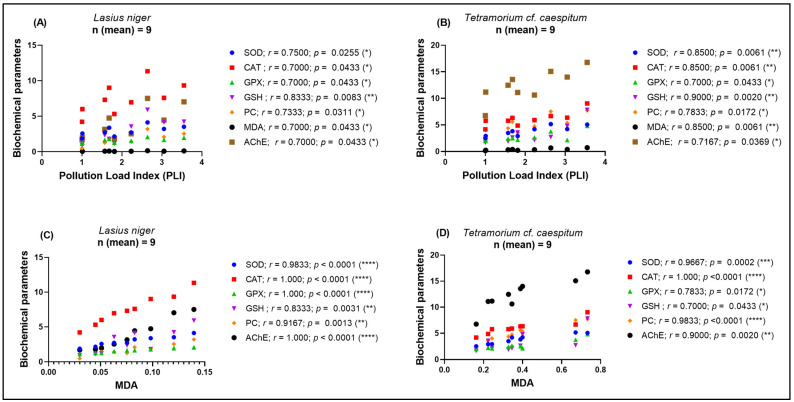
Spearman correlation analysis between Pollution Load Index (*PLI*), malondialdehyde (MDA), and biochemical biomarkers in *L. niger* and *T.* cf. *caespitum* (**A**) Correlations between *PLI* and biochemical parameters in *L. niger*; (**B**) correlations between *PLI* and biochemical parameters in *T.* cf. *caespitum*; (**C**) correlations between MDA and biochemical parameters in *L. niger*; and (**D**) correlations between MDA and biochemical parameters in *T.* cf. *caespitum*. Correlations were calculated using site-level mean values. CM1–CM8 represent sampling sites from the Copșa Mică industrial area, and TAPU represents the local low-contamination reference site within the study landscape. Complete Spearman correlation coefficients and corresponding *p* values are provided in Table 5. Significance coding: * *p* < 0.05; ** *p* < 0.01; *** *p* < 0.001; **** *p* < 0.0001.

Spearman correlations were calculated between *PLI* and site-level mean biomarker values for each species. Complete correlation coefficients and corresponding *p* values are presented in Table 5 and illustrated in Figure 7A,B. Because these analyses were based on site-level mean values, the number of independent observations was limited, and the correlations were interpreted as patterns of covariation.

In both taxa, *PLI* was positively correlated with all measured biomarkers, including antioxidant enzymes, GSH, oxidative damage markers, and AChE activity (Figure 7A,B; Table 5). The coefficients were generally higher in *T.* cf. *caespitum* for SOD, CAT, GSH, and MDA, while *L. niger* also showed significant positive associations across all biomarkers.

Spearman correlations were also calculated between MDA and the other biomarkers using site-level mean values. In both taxa, MDA was positively correlated with antioxidant enzymes, GSH, PC, and AChE activity (Figure 7C,D; Table 5). The strongest associations, including perfect monotonic relationships observed for some marker pairs, should be interpreted cautiously because they were based on a limited number of site-level observations.

Overall, the correlation analyses describe coordinated covariation among soil contamination, oxidative damage markers, antioxidant biomarkers, and AChE activity across sites.

## 4. Discussion

### 4.1. Metal(loid) Contamination Gradient and Biological Relevance

Metal(loid) contamination represents one of the most persistent anthropogenic pressures on terrestrial ecosystems because potentially toxic elements are non-biodegradable, can remain environmentally available over long periods, and may exert chronic sublethal effects on soil-associated biota [12,38,39]. Under field conditions, organisms are rarely exposed to a single element, but rather, to complex mixtures that may converge on common toxicological pathways, particularly oxidative imbalance and neurophysiological disruption [12,39].

The Copșa Mică area should therefore be interpreted as a relative contamination gradient within a historically impacted landscape, rather than as a simple polluted-versus-pristine contrast [16]. The Pollution Load Index (*PLI*), remained above 1 at all Copșa Mică sites when calculated using the full 14-element panel, indicating persistent metal(loid) pressure above the local TAPU baseline throughout the impacted area [16]. When the *PLI* was recalculated using the seven toxicologically prioritized elements, namely Pb, Cd, Zn, Cu, Ni, As, and Hg, the contamination gradient became more pronounced, with the highest values recorded at CM5, CM4, and CM1. This stronger separation suggests that site differences were driven not only by total elemental load, but also by enrichment in metals with higher relevance for oxidative stress and neuroenzymatic disturbance [12,39,40,41].

The threshold-based classification further reinforces the environmental relevance of this gradient. Arsenic exceeded the intervention threshold at all sites, including TAPU, while cadmium exceeded the intervention threshold at most Copșa Mică sites, zinc reached intervention levels at CM1, CM4, and CM5, and lead frequently exceeded the alert threshold in the industrial area [23]. These findings show that contamination was environmentally substantial. However, because TAPU also showed exceedances, it should be considered a local low-contamination reference site rather than an uncontaminated control, a distinction important for avoiding overinterpretation of site contrasts [42,43]. Mitochondria are among the major intracellular targets of these processes, as metals can interfere with electron transport, ATP production, and ROS control, thereby providing a biologically plausible framework for the coordinated variation observed here between antioxidant biomarkers (SOD, CAT, GPx, and GSH) and damage markers (MDA and PC) [42,43,44]. In addition, many metals show high affinity for sulfhydryl groups, which may alter the activity of redox-sensitive proteins and enzymes, including systems involved in neurotransmission [45,46]. Within this framework, field-associated changes in AChE activity may reflect not only direct neurochemical perturbation, but also secondary redox-mediated modulation of enzymatic regulation [47].

Field studies also support the use of ants and their nest matrices as indicators of spatial metal contamination [13,15,16,40,48,49,50]. Overall, the metal(loid) profile identified in Copșa Mică appears ecotoxicologically relevant for soil-associated social insects. Nevertheless, because this was a field-based observational study without direct tissue bioaccumulation measurements, the observed responses should be interpreted as biologically meaningful associations along a contamination gradient rather than as definitive evidence of metal-specific causality.

### 4.2. Oxidative Stress as the Central Mechanistic Axis

Oxidative stress represents the central mechanistic axis of metal(loid)-induced toxicity in invertebrates, including terrestrial insects. Reactive oxygen species (ROS) constitute the shared point of convergence of metal effects, regardless of whether metals are redox-active or induce oxidative stress indirectly [12,38,39]. Transition metals can participate in Fenton- and Haber–Weiss-type reactions, while Cd and Pb can promote ROS formation indirectly, supporting the interpretation of oxidative imbalance as a major pathway in contaminant toxicity [38,39,41,51,52]. In insects, oxidative stress also has an ecophysiological dimension, acting not only as a damage mechanism but also as a signaling node in responses to multiple stressors [53].

The increased SOD, CAT, and GPx activities observed at several high-*PLI* sites are consistent with antioxidant activation under chronic contaminant pressure. Previous experimental work supports this interpretation: Cd exposure can disrupt mitochondrial function and increase ROS production [40,54], while chronic exposure in insects has been associated with increased antioxidant enzyme activity and lipid peroxidation [40]. These observations are consistent with the pattern identified in the present study, where increased SOD activity at high-*PLI* sites suggests intensified superoxide production as an early step in the oxidative cascade. Similar coordinated activation of SOD, CAT, and GPx, together with oxidative damage and metabolic disruption, has also been reported in metal-exposed insect models [14].

More broadly, studies across insect taxa indicate that antioxidant responses often increase with contaminant burden until compensatory capacity is exceeded and oxidative damage emerges [55,56]. This aligns with recent literature describing oxidative stress as a mediator of disrupted cellular signaling, inflammatory pathways, and broader adaptive responses [57,58], including processes relevant to social insects such as longevity, behavior, and neurotransmission [13,53,55].

The glutathione system further supports this integrated redox interpretation. GSH contributes to metal chelation, thiol buffering, and peroxide neutralization through GPx-dependent reactions [59]. Metals with affinity for sulfhydryl groups can alter thiol redox balance and disrupt redox-sensitive proteins [58,60], while the thioredoxin system also contributes to redox proteostasis and adaptive signaling beyond classical antioxidant defense [60]. Ecotoxicological observations in social insects further support links between contaminant exposure, oxidative stress, and behavioral or physiological impairment [54], and insects have been proposed as useful oxidative stress sentinels because redox biomarkers integrate contaminant pressure into measurable biological signals [13,55,61].

In our study, increased GSH levels at sites with elevated *PLI* suggest mobilization of thiol reserves for detoxification. However, the concomitant accumulation of MDA and PC indicates that antioxidant mechanisms, although activated, were not fully protective, a pattern consistent with compensated oxidative stress (defensive upregulation coupled with residual molecular damage) [61,62]. The significant associations between *PLI* and redox biomarkers, together with the tight association between MDA and AChE, suggest that oxidative responses may extend beyond metabolic compartments into neuroenzymatic regulation. Metals are well-documented redox-active neurotoxicants capable of inducing mitochondrial dysfunction, thiol interactions, and oxidative modification of neuronal proteins [13,38], while lipid peroxidation and protein carbonylation are established indicators of oxidative damage in metal-exposed insects [63].

Although AChE inhibition is classically associated with organophosphate exposure, metals may also modulate cholinesterase activity through redox-mediated structural and regulatory mechanisms [22]. Therefore, the positive association between MDA and AChE activity observed here may reflect redox-linked neuroenzymatic modulation rather than direct enzymatic inhibition. Overall, the coordinated variation observed in L. niger and T. cf. caespitum at high-*PLI* sites is biologically plausible and consistent with the experimental literature.

### 4.3. Ecological Significance

In social insects, individual-level physiological changes may have broader ecological relevance because colony functioning depends on the cumulative performance of workers. Longevity, foraging efficiency, defense, and reproductive success are linked to worker physiological status [57] and oxidative stress induced by metal(loid) contamination may therefore have implications beyond the cellular level [51].

Redox balance has been associated with longevity, division of labor, and worker lifespan in ants and bees [62,64]. For example, redox parameters have been linked to behavioral transitions and longevity in *Harpegnathos saltator* [62], while oxidative stress can influence foraging performance and susceptibility to additional stressors in *Apis mellifera* [65,66,67]. Social insects are also vulnerable to multiple stressors, and cumulative oxidative stress may amplify energetic costs associated with activities such as foraging and thermoregulation [57,68]. Broader syntheses suggest that redox imbalance can impair individual performance and potentially affect colony demographic stability [56].

In the present study, antioxidant activation together with MDA and PC accumulation suggests possible sublethal physiological costs at sites with elevated *PLI*. Although colony-level performance was not directly assessed, persistent oxidative damage and altered AChE activity may indicate reduced physiological resilience under chronic exposure. Because ants contribute to soil dynamics, seed dispersal, and regulation of invertebrate communities [69], such biomarker changes may serve as early indicators of broader ecological disturbance. Thus, the results support the view that metal(loid)-associated oxidative stress has relevance not only at the individual level, but potentially at colony and ecosystem levels, in line with literature on physiological trade-offs and social-insect vulnerability to anthropogenic stressors [55,56,57,68].

### 4.4. Limitations and Future Directions

Several limitations should be considered. First, this was an observational field study conducted in a naturally heterogeneous environment. Although the geochemical profile and *PLI*–biomarker associations support the biological relevance of the contamination gradient, oxidative and neuroenzymatic biomarkers may also be influenced by microclimatic variation, trophic resource availability, colony demography, pathogens, parasites, and soil-associated microorganisms. Therefore, the present results should be interpreted as field-based associations rather than proof of a single-cause mechanism.

Second, exposure was inferred from soil metal(loid) concentrations and *PLI*, whereas internal metal burdens were not measured directly in ant tissues. This distinction is critical because antioxidant and detoxification responses are expected to depend more directly on internalized dose than on total soil concentrations. Previous studies show that insects differ in metal uptake, transfer, and body burdens, and that accumulation patterns vary among taxa, tissues, and exposure routes. In ants, selective metal accumulation and tissue-specific sequestration have been documented; therefore, soil contamination alone cannot fully predict internal dose or biochemical response intensity [20].

Third, TAPU should be considered a local low-contamination reference site rather than pristine control, because it also showed exceedances, particularly for arsenic and selenium. Fourth, the biological replication structure limits the strength of quantitative inference: three colonies were sampled per site, and the main correlation analyses were performed on site means. This design was appropriate for evaluating monotonic pressure–response relationships at the site level, but the limited number of independent site-level observations reduces statistical power and may accentuate the strength of some correlations. Accordingly, the strongest associations should be interpreted as coordinated covariation rather than definitive estimates of effect magnitude.

Finally, the biomarker panel provides mechanistic information on oxidative and neuroenzymatic status, but it does not directly quantify functional ecological consequences such as foraging, survival, brood development, colony performance, or ecosystem functioning. Future work should therefore combine direct tissue bioaccumulation measurements with species-specific bioaccumulation factors, controlled exposure experiments, and behavioral or colony-level endpoints. Such integration would strengthen causal inference and improve the interpretation of ants as biomonitoring models in metal(loid)-impacted terrestrial ecosystems.

## 5. Conclusions

This study suggests that the Copșa Mică area represents a biologically relevant gradient of cumulative soil metal(oid) contamination, and that increasing contamination pressure is associated with coordinated oxidative and neuroenzymatic responses in *L. niger* and *T.* cf. *caespitum*. Higher *PLI* values were consistently linked to increased antioxidant activity, elevated glutathione, greater oxidative damage, and altered AChE activity, supporting a pattern of coordinated covariation between soil contamination and physiological responses under field conditions.

These findings support the potential use of ants as biomonitoring organisms for detecting biological responses along metal(loid)-impacted soil gradients. However, because this was a field-based study using a local low-contamination reference site, site-level correlation analyses, and no direct tissue bioaccumulation measurements, the results should be interpreted as field-based associations rather than definitive proof of metal-specific causality.

Overall, the study supports the use of integrated redox and neuroenzymatic biomarkers for assessing sublethal stress in metal(oid)-impacted soils and provides a basis for future work combining geochemical data, tissue bioaccumulation measurements, controlled exposure experiments, and functional ecological endpoints.

## Figures and Tables

**Table 1:** Instrumental (a) and data acquisition (b) parameters of ICP-MS.

Instrumental Parameters	Value	Data Acquisition Parameters for Quantitative Mode	Value
RF power	1.4 kW	Measuring mode	Peak hopping
Argon gas flow		Point per peak	3
Nebulizer	1. L/min	Scans/Replicate	10
Plasma	18.0 L/min	Replicate/Sample	10
Lens voltage		Dwell time (ms)	1
Mirror lens left	37 V		
Mirror lens right	31 V		
Mirror lens bottom	30 V		
Sample uptake rate	40 s	Integration time (s)	395.08 s

**Table 2:** Microwave digestion program used for soil sample extraction with the Mars 5 Microwave System.

Step	Power (Level)		Ramp Time (min)	Pressure (psi)	Temperature (°C)	Hold Time (min)
1	800	100%	6	800	200	20
2	15 min cooling					

**Table 3:** Romanian regulatory thresholds for selected soil elements according to Order No. 756/1997 [26] (mg/kg dry weight).

Element	Normal Value	Alert Threshold (Sensitive Use)	Alert Threshold (Less Sensitive Use)	Intervention Threshold (Sensitive Use)	Intervention Threshold (Less Sensitive Use)
As	5	15	25	25	50
Cd	1	3	5	5	10
Co	15	30	100	50	250
Cr total	30	100	300	300	600
Cu	20	100	250	200	500
Mn	900	1500	2000	2500	4000
Hg	0.1	1	4	2	10
Ni	20	75	200	150	500
Pb	20	50	250	100	1000
Zn	100	300	700	600	1500

**Table 4:** Classification of soil metal(loid)exceedances according to Order 756/1997 [26]: N = normal, A = alert, I = intervention.

Site	As	Cd	Zn	Pb	Cu	Ni	Cr	Co	Se	Hg
CM1	I	I	I	A	N	N	N	N	I	N
CM2	I	I	A	A	N	N	N	N	I	N
CM3	I	I	A	A	N	N	N	N	I	N
CM4	I	I	I	A	N	N	N	N	I	N
CM5	I	I	I	A	N	N	N	N	I	N
CM6	I	I	A	N	N	N	N	N	I	N
CM7	I	I	A	A	N	N	N	N	N	N
CM8	I	N	N	N	N	N	N	N	I	N
TAPU	I	N	N	N	N	N	N	I	I	N

**Table 5:** Spearman correlation coefficients between *PLI*, MDA, and biochemical biomarkers in *Lasius niger* and *Tetramorium* cf. *caespitum*.

*Species*	*Predictor*	*Biomarker*	*Spearman r*	*p Value*	*Significance*
*L. niger*	*PLI*	SOD	0.7500	0.0255	*
*L. niger*	*PLI*	CAT	0.7000	0.0433	*
*L. niger*	*PLI*	GPx	0.7000	0.0433	*
*L. niger*	*PLI*	GSH	0.8333	0.0083	**
*L. niger*	*PLI*	PC	0.7333	0.0311	*
*L. niger*	*PLI*	MDA	0.7000	0.0433	*
*L. niger*	*PLI*	AChE	0.7000	0.0433	*
*T.* cf. *caespitum*	*PLI*	SOD	0.8500	0.0061	**
*T.* cf. *caespitum*	*PLI*	CAT	0.8500	0.0061	**
*T.* cf. *caespitum*	*PLI*	GPx	0.7000	0.0433	*
*T.* cf. *caespitum*	*PLI*	GSH	0.9000	0.0020	**
*T.* cf. *caespitum*	*PLI*	PC	0.7833	0.0172	*
*T.* cf. *caespitum*	*PLI*	MDA	0.8500	0.0061	**
*T.* cf. *caespitum*	*PLI*	AChE	0.7167	0.0369	*
*L. niger*	MDA	SOD	0.9833	<0.0001	****
*L. niger*	MDA	CAT	1.0000	<0.0001	****
*L. niger*	MDA	GPx	1.0000	<0.0001	****
*L. niger*	MDA	GSH	0.8833	0.0031	**
*L. niger*	MDA	PC	0.9167	0.0013	**
*L. niger*	MDA	AChE	1.0000	<0.0001	****
*T.* cf. *caespitum*	MDA	SOD	0.9667	0.0002	***
*T.* cf. *caespitum*	MDA	CAT	1.0000	<0.0001	****
*T.* cf. *caespitum*	MDA	GPx	0.7833	0.0172	*
*T.* cf. *caespitum*	MDA	GSH	0.7000	0.0433	*
*T.* cf. *caespitum*	MDA	PC	0.9833	<0.0001	****
*T.* cf. *caespitum*	MDA	AChE	0.9000	0.0020	**

Note: *PLI*, Pollution Load Index; SOD, superoxide dismutase; CAT, catalase; GPx, glutathione peroxidase; GSH, reduced glutathione; PC, protein carbonyls; MDA, malondialdehyde; AChE, acetylcholinesterase. Correlations were calculated using site-level mean values. Spearman’s rank correlation coefficients and two-tailed *p* values are reported. Significance coding: * *p* < 0.05; ** *p* < 0.01; *** *p* < 0.001; **** *p* < 0.0001.

## Data Availability

The data presented in this study are available in this article.

## Supplementary Materials

The following supporting information can be downloaded at: https://www.mdpi.com/article/10.3390/life16050743/s1. Table S1: Tukey post hoc comparisons versus the TAPU reference site for antioxidant enzyme activities in *L. niger* and *T.* cf. *caespitum*; Table S2: Tukey post hoc comparisons versus the TAPU reference site for reduced glutathione levels in *L. niger* and *T.* cf. *caespitum*; Table S3: Tukey post hoc comparisons versus the TAPU reference site for oxidative damage biomarkers in *L. niger* and *T.* cf. *caespitum*; Table S4: Tukey post hoc comparisons versus the TAPU reference site for AChE activity in *L. niger* and *T.* cf. *caespitum*.

## Abbreviations

The following abbreviations are used in this manuscript:

AChE	Acetylcholinesterase
As	Arsenic
ATP	Adenosine triphosphate
CAT	Catalase
Cd	Cadmium
CM	Copșa Mică
Cu	Copper
Fe	Iron
GPx	Glutathione peroxidase
GSH	Reduced glutathione
Hg	Mercury
MDA	Malondialdehyde
Mn	Manganese
Ni	Nickel
Pb	Lead
PC	Protein carbonyls
*PLI*	Pollution Load Index
ROS	Reactive oxygen species
SH	Sulfhydryl groups
SOD	Superoxide dismutase
TAPU	Reference site (Țapu)
Zn	Zinc
