# Supplementary material for: Cumulative Soil Metal Contamination Reshapes Oxidative and Neuroenzymatic Stress Responses in Ants Across an Industrial Pollution Gradient
Source: Life (Basel). 2026 Apr 29;16(5):743. doi: 10.3390/life16050743 (PMC13208561; doi:10.3390/life16050743)
Supplement: Supplementary file 1 [file life-16-00743-s001.zip › life-4270227-supplementary.pdf]

**Table S1.** Tukey post hoc comparisons versus the TAPU reference site for antioxidant enzyme activities in *Lasius niger* and *Tetramorium cf. caespitum*

| Biomarker | Species | Site vs. TAPU | Mean difference | 95% CI of difference | Adjusted p value | Significance |
|---|---|---|---|---|---|---|
| **SOD** | *L. niger* | CM1 vs. TAPU | 2.258 | 1.587 to 2.929 | <0.0001 | **** |
| **SOD** | *L. niger* | CM2 vs. TAPU | 0.8449 | 0.1743 to 1.515 | 0.0041 | ** |
| **SOD** | *L. niger* | CM3 vs. TAPU | 0.8454 | 0.1749 to 1.516 | 0.0041 | ** |
| **SOD** | *L. niger* | CM4 vs. TAPU | 1.348 | 0.6779 to 2.019 | <0.0001 | **** |
| **SOD** | *L. niger* | CM5 vs. TAPU | 1.642 | 0.9711 to 2.312 | <0.0001 | **** |
| **SOD** | *L. niger* | CM6 vs. TAPU | 1.504 | 0.8333 to 2.174 | <0.0001 | **** |
| **SOD** | *L. niger* | CM7 vs. TAPU | 0.2878 | −0.3828 to 0.9583 | 0.9044 | ns |
| **SOD** | *L. niger* | CM8 vs. TAPU | 0.6979 | 0.02738 to 1.368 | 0.0350 | * |
| **SOD** | *T. cf. caespitum* | CM1 vs. TAPU | 2.644 | 1.018 to 4.271 | <0.0001 | **** |
| **SOD** | *T. cf. caespitum* | CM2 vs. TAPU | 0.9862 | −0.6400 to 2.612 | 0.5894 | ns |
| **SOD** | *T. cf. caespitum* | CM3 vs. TAPU | 1.676 | 0.04934 to 3.302 | 0.0385 | * |
| **SOD** | *T. cf. caespitum* | CM4 vs. TAPU | 1.709 | 0.08248 to 3.335 | 0.0321 | * |
| **SOD** | *T. cf. caespitum* | CM5 vs. TAPU | 2.567 | 0.9404 to 4.193 | 0.0001 | *** |
| **SOD** | *T. cf. caespitum* | CM6 vs. TAPU | 1.314 | −0.3124 to 2.940 | 0.2118 | ns |
| **SOD** | *T. cf. caespitum* | CM7 vs. TAPU | 0.4026 | −1.224 to 2.029 | 0.9968 | ns |
| **SOD** | *T. cf. caespitum* | CM8 vs. TAPU | 0.4053 | −1.221 to 2.032 | 0.9966 | ns |
| **CAT** | *L. niger* | CM1 vs. TAPU | 7.456 | 5.425 to 9.486 | <0.0001 | **** |
| **CAT** | *L. niger* | CM2 vs. TAPU | 3.099 | 1.068 to 5.130 | 0.0002 | *** |
| **CAT** | *L. niger* | CM3 vs. TAPU | 2.753 | 0.7222 to 4.784 | 0.0015 | ** |
| **CAT** | *L. niger* | CM4 vs. TAPU | 3.376 | 1.345 to 5.407 | <0.0001 | **** |
| **CAT** | *L. niger* | CM5 vs. TAPU | 5.134 | 3.103 to 7.165 | <0.0001 | **** |
| **CAT** | *L. niger* | CM6 vs. TAPU | 4.805 | 2.774 to 6.836 | <0.0001 | **** |
| **CAT** | *L. niger* | CM7 vs. TAPU | 1.097 | −0.9334 to 3.128 | 0.7274 | ns |
| **CAT** | *L. niger* | CM8 vs. TAPU | 1.790 | −0.2410 to 3.821 | 0.1279 | ns |
| **CAT** | *T. cf. caespitum* | CM1 vs. TAPU | 2.514 | 0.03318 to 4.994 | 0.0446 | * |

| | | | | | | |
|---|---|---|---|---|---|---|
| **CAT** | *T. cf. caespitum* | CM2 vs. TAPU | 1.621 | −0.8592 to 4.102 | 0.4880 | ns |
| **CAT** | *T. cf. caespitum* | CM3 vs. TAPU | 1.742 | −0.7382 to 4.223 | 0.3882 | ns |
| **CAT** | *T. cf. caespitum* | CM4 vs. TAPU | 2.190 | −0.2909 to 4.670 | 0.1267 | ns |
| **CAT** | *T. cf. caespitum* | CM5 vs. TAPU | 4.871 | 2.391 to 7.352 | <0.0001 | **** |
| **CAT** | *T. cf. caespitum* | CM6 vs. TAPU | 2.159 | −0.3217 to 4.639 | 0.1386 | ns |
| **CAT** | *T. cf. caespitum* | CM7 vs. TAPU | 0.7149 | −1.765 to 3.195 | 0.9910 | ns |
| **CAT** | *T. cf. caespitum* | CM8 vs. TAPU | 1.620 | −0.8605 to 4.100 | 0.4891 | ns |
| **GPx** | *L. niger* | CM1 vs. TAPU | 0.9707 | 0.5553 to 1.386 | <0.0001 | **** |
| **GPx** | *L. niger* | CM2 vs. TAPU | 0.5471 | 0.1317 to 0.9625 | 0.0022 | ** |
| **GPx** | *L. niger* | CM3 vs. TAPU | 0.4191 | 0.003678 to 0.8344 | 0.0464 | * |
| **GPx** | *L. niger* | CM4 vs. TAPU | 0.5571 | 0.1417 to 0.9725 | 0.0017 | ** |
| **GPx** | *L. niger* | CM5 vs. TAPU | 0.8527 | 0.4374 to 1.268 | <0.0001 | **** |
| **GPx** | *L. niger* | CM6 vs. TAPU | 0.6970 | 0.2816 to 1.112 | <0.0001 | **** |
| **GPx** | *L. niger* | CM7 vs. TAPU | 0.1646 | −0.2508 to 0.5800 | 0.9375 | ns |
| **GPx** | *L. niger* | CM8 vs. TAPU | 0.1779 | −0.2375 to 0.5933 | 0.9055 | ns |
| **GPx** | *T. cf. caespitum* | CM1 vs. TAPU | 1.759 | 0.8531 to 2.664 | <0.0001 | **** |
| **GPx** | *T. cf. caespitum* | CM2 vs. TAPU | 0.4276 | −0.4781 to 1.333 | 0.8473 | ns |
| **GPx** | *T. cf. caespitum* | CM3 vs. TAPU | 0.6181 | −0.2876 to 1.524 | 0.4279 | ns |
| **GPx** | *T. cf. caespitum* | CM4 vs. TAPU | 0.1543 | −0.7514 to 1.060 | 0.9998 | ns |
| **GPx** | *T. cf. caespitum* | CM5 vs. TAPU | 2.829 | 1.923 to 3.735 | <0.0001 | **** |
| **GPx** | *T. cf. caespitum* | CM6 vs. TAPU | 0.5036 | −0.4021 to 1.409 | 0.6959 | ns |
| **GPx** | *T. cf. caespitum* | CM7 vs. TAPU | 0.2236 | −0.6821 to 1.129 | 0.9968 | ns |
| **GPx** | *T. cf. caespitum* | CM8 vs. TAPU | 0.1467 | −0.7590 to 1.052 | 0.9999 | ns |

**Note:** SOD, superoxide dismutase; CAT, catalase; GPx, glutathione peroxidase; TAPU, local reference site. Values are Tukey-adjusted post hoc comparisons versus TAPU. Significance coding: ns, not significant; * $p < 0.05$; ** $p < 0.01$; *** $p < 0.001$; **** $p < 0.0001$.

**Table S2**. Tukey post hoc comparisons versus the TAPU reference site for reduced glutathione levels in *Lasius niger* and *Tetramorium cf. caespitum*

| Biomarker | Species | Site vs. TAPU | Mean difference | 95% CI of difference | Adjusted p value | Significance |
|---|---|---|---|---|---|---|
| **GSH** | *L. niger* | CM1 vs. TAPU | 4.959 | 3.599 to 6.318 | <0.0001 | **** |
| **GSH** | *L. niger* | CM2 vs. TAPU | 1.383 | 0.02321 to 2.743 | 0.0432 | * |
| **GSH** | *L. niger* | CM3 vs. TAPU | 2.598 | 1.239 to 3.958 | <0.0001 | **** |
| **GSH** | *L. niger* | CM4 vs. TAPU | 3.182 | 1.822 to 4.541 | <0.0001 | **** |
| **GSH** | *L. niger* | CM5 vs. TAPU | 3.281 | 1.921 to 4.640 | <0.0001 | **** |
| **GSH** | *L. niger* | CM6 vs. TAPU | 0.8388 | −0.5209 to 2.199 | 0.5668 | ns |
| **GSH** | *L. niger* | CM7 vs. TAPU | 0.2034 | −1.156 to 1.563 | >0.9999 | ns |
| **GSH** | *L. niger* | CM8 vs. TAPU | 0.2563 | −1.103 to 1.616 | 0.9995 | ns |
| **GSH** | *T. cf. caespitum* | CM1 vs. TAPU | 1.054 | −0.8183 to 2.927 | 0.6817 | ns |
| **GSH** | *T. cf. caespitum* | CM2 vs. TAPU | 0.2528 | −1.620 to 2.125 | >0.9999 | ns |
| **GSH** | *T. cf. caespitum* | CM3 vs. TAPU | 0.5034 | −1.369 to 2.376 | 0.9943 | ns |
| **GSH** | *T. cf. caespitum* | CM4 vs. TAPU | 3.131 | 1.259 to 5.003 | <0.0001 | **** |
| **GSH** | *T. cf. caespitum* | CM5 vs. TAPU | 6.162 | 4.290 to 8.035 | <0.0001 | **** |
| **GSH** | *T. cf. caespitum* | CM6 vs. TAPU | 0.9469 | −0.9256 to 2.819 | 0.7923 | ns |
| **GSH** | *T. cf. caespitum* | CM7 vs. TAPU | 1.906 | 0.03344 to 3.778 | 0.0429 | * |
| **GSH** | *T. cf. caespitum* | CM8 vs. TAPU | 0.4202 | −1.452 to 2.293 | 0.9984 | ns |

**Note:** GSH, reduced glutathione; TAPU, local reference site. Values are Tukey-adjusted post hoc comparisons versus TAPU. Significance coding: ns, not significant; * $p < 0.05$; ** $p < 0.01$; *** $p < 0.001$; **** $p < 0.0001$.

**Table S3.** Tukey post hoc comparisons versus the TAPU reference site for oxidative damage biomarkers in *Lasius niger* and *Tetramorium cf. caespitum*

| Biomarker | Species | Site vs. TAPU | Mean difference | 95% CI of difference | Adjusted p value | Significance |
|---|---|---|---|---|---|---|
| **PC** | *L. niger* | CM1 vs. TAPU | 2.696 | 1.933 to 3.458 | <0.0001 | **** |
| **PC** | *L. niger* | CM2 vs. TAPU | 0.7747 | 0.01243 to 1.537 | 0.0435 | * |
| **PC** | *L. niger* | CM3 vs. TAPU | 0.9367 | 0.1744 to 1.699 | 0.0057 | ** |
| **PC** | *L. niger* | CM4 vs. TAPU | 1.595 | 0.8329 to 2.358 | <0.0001 | **** |
| **PC** | *L. niger* | CM5 vs. TAPU | 2.044 | 1.282 to 2.806 | <0.0001 | **** |
| **PC** | *L. niger* | CM6 vs. TAPU | 1.390 | 0.6275 to 2.152 | <0.0001 | **** |

| | | | | | | |
|---|---|---|---|---|---|---|
| **PC** | *L. niger* | CM7 vs. TAPU | 0.6300 | −0.1323 to 1.392 | 0.1876 | ns |
| **PC** | *L. niger* | CM8 vs. TAPU | 0.9541 | 0.1918 to 1.716 | 0.0045 | ** |
| **PC** | *T. cf. caespitum* | CM1 vs. TAPU | 4.958 | 3.213 to 6.704 | <0.0001 | **** |
| **PC** | *T. cf. caespitum* | CM2 vs. TAPU | 1.824 | 0.07854 to 3.570 | 0.0338 | * |
| **PC** | *T. cf. caespitum* | CM3 vs. TAPU | 2.176 | 0.4304 to 3.922 | 0.0047 | ** |
| **PC** | *T. cf. caespitum* | CM4 vs. TAPU | 2.807 | 1.061 to 4.553 | <0.0001 | **** |
| **PC** | *T. cf. caespitum* | CM5 vs. TAPU | 5.165 | 3.420 to 6.911 | <0.0001 | **** |
| **PC** | *T. cf. caespitum* | CM6 vs. TAPU | 3.017 | 1.272 to 4.763 | <0.0001 | **** |
| **PC** | *T. cf. caespitum* | CM7 vs. TAPU | 0.9953 | −0.7505 to 2.741 | 0.6669 | ns |
| **PC** | *T. cf. caespitum* | CM8 vs. TAPU | 1.458 | −0.2878 to 3.204 | 0.1770 | ns |
| **MDA** | *L. niger* | CM1 vs. TAPU | 0.1097 | 0.08223 to 0.1372 | <0.0001 | **** |
| **MDA** | *L. niger* | CM2 vs. TAPU | 0.04548 | 0.01802 to 0.07294 | <0.0001 | **** |
| **MDA** | *L. niger* | CM3 vs. TAPU | 0.03303 | 0.005566 to 0.06049 | 0.0075 | ** |
| **MDA** | *L. niger* | CM4 vs. TAPU | 0.05277 | 0.02531 to 0.08024 | <0.0001 | **** |
| **MDA** | *L. niger* | CM5 vs. TAPU | 0.09045 | 0.06299 to 0.1179 | <0.0001 | **** |
| **MDA** | *L. niger* | CM6 vs. TAPU | 0.06825 | 0.04078 to 0.09571 | <0.0001 | **** |
| **MDA** | *L. niger* | CM7 vs. TAPU | 0.01514 | −0.01232 to 0.04260 | 0.7057 | ns |
| **MDA** | *L. niger* | CM8 vs. TAPU | 0.02094 | −0.006520 to 0.04840 | 0.2797 | ns |
| **MDA** | *T. cf. caespitum* | CM1 vs. TAPU | 0.5093 | 0.3865 to 0.6321 | <0.0001 | **** |
| **MDA** | *T. cf. caespitum* | CM2 vs. TAPU | 0.1652 | 0.04234 to 0.2880 | 0.0017 | ** |
| **MDA** | *T. cf. caespitum* | CM3 vs. TAPU | 0.1828 | 0.05995 to 0.3056 | 0.0003 | *** |
| **MDA** | *T. cf. caespitum* | CM4 vs. TAPU | 0.2372 | 0.1144 to 0.3601 | <0.0001 | **** |
| **MDA** | *T. cf. caespitum* | CM5 vs. TAPU | 0.5702 | 0.4474 to 0.6931 | <0.0001 | **** |
| **MDA** | *T. cf. caespitum* | CM6 vs. TAPU | 0.2264 | 0.1036 to 0.3492 | <0.0001 | **** |
| **MDA** | *T. cf. caespitum* | CM7 vs. TAPU | 0.06052 | −0.06231 to 0.1834 | 0.8146 | ns |
| **MDA** | *T. cf. caespitum* | CM8 vs. TAPU | 0.08069 | −0.04215 to 0.2035 | 0.4810 | ns |

**Note:** PC, protein carbonyls; MDA, malondialdehyde; TAPU, local reference site. Values represent Tukey-adjusted post hoc comparisons versus TAPU. Significance coding: ns, not significant; * $p < 0.05$; ** $p < 0.01$; *** $p < 0.001$; **** $p < 0.0001$.

**Table S4**. Tukey post hoc comparisons versus the TAPU reference site for acetylcholinesterase activity in *Lasius niger* and *Tetramorium cf. caespitum*

| Biomarker | Species | Site vs. TAPU | Mean difference | 95% CI of difference | Adjusted p value | Significance |
|---|---|---|---|---|---|---|
| **AChE** | *L. niger* | CM1 vs. TAPU | 5.807 | 4.215 to 7.399 | <0.0001 | **** |
| **AChE** | *L. niger* | CM2 vs. TAPU | 1.448 | −0.1440 to 3.040 | 0.1037 | ns |
| **AChE** | *L. niger* | CM3 vs. TAPU | 0.8234 | −0.7687 to 2.416 | 0.7716 | ns |
| **AChE** | *L. niger* | CM4 vs. TAPU | 2.743 | 1.151 to 4.335 | <0.0001 | **** |
| **AChE** | *L. niger* | CM5 vs. TAPU | 5.328 | 3.736 to 6.920 | <0.0001 | **** |
| **AChE** | *L. niger* | CM6 vs. TAPU | 3.033 | 1.441 to 4.625 | <0.0001 | **** |
| **AChE** | *L. niger* | CM7 vs. TAPU | 0.1116 | −1.481 to 1.704 | >0.9999 | ns |
| **AChE** | *L. niger* | CM8 vs. TAPU | 0.2710 | −1.321 to 1.863 | 0.9998 | ns |
| **AChE** | *T. cf. caespitum* | CM1 vs. TAPU | 8.304 | 4.750 to 11.86 | <0.0001 | **** |
| **AChE** | *T. cf. caespitum* | CM2 vs. TAPU | 5.712 | 2.158 to 9.266 | <0.0001 | **** |
| **AChE** | *T. cf. caespitum* | CM3 vs. TAPU | 3.863 | 0.3094 to 7.417 | 0.0230 | * |
| **AChE** | *T. cf. caespitum* | CM4 vs. TAPU | 7.241 | 3.687 to 10.80 | <0.0001 | **** |
| **AChE** | *T. cf. caespitum* | CM5 vs. TAPU | 10.02 | 6.463 to 13.57 | <0.0001 | **** |
| **AChE** | *T. cf. caespitum* | CM6 vs. TAPU | 6.790 | 3.236 to 10.34 | <0.0001 | **** |
| **AChE** | *T. cf. caespitum* | CM7 vs. TAPU | 4.339 | 0.7852 to 7.893 | 0.0062 | ** |
| **AChE** | *T. cf. caespitum* | CM8 vs. TAPU | 4.407 | 0.8530 to 7.961 | 0.0051 | ** |

**Note:** AChE, acetylcholinesterase; TAPU, local reference site. Values represent Tukey-adjusted post hoc comparisons versus TAPU. Significance coding: ns, not significant; * $p < 0.05$; ** $p < 0.01$; *** $p < 0.001$; **** $p < 0.0001$.
